# ZNF32 promotes the self-renewal of colorectal cancer cells by regulating the LEPR-STAT3 signaling pathway

**DOI:** 10.1038/s41419-022-04530-4

**Published:** 2022-02-03

**Authors:** Jun Li, Xiaoan Li, Lili Lan, Lin Sun, Xuemei Li, Yaqi Li, Yan Tian, Tongqin Zhang, Yan Zhou, Chunfen Mo, Xiangsheng Fu

**Affiliations:** 1 Department of Gastroenterology, Clinical medical college and the first affiliated hospital of Chengdu medical college, Chengdu, China; 2 Department of Gastroenterology, Mianyang central hospital, Mianyang, China; 3 Department of Immunology, School of Basic Medical Sciences, Chengdu Medical College, Chengdu, China

**Keywords:** Cancer stem cells, Colon cancer, Cancer stem cells

## Abstract

Due to the self-renewal characteristics and tumorigenic abilities of cancer stem cells (CSCs), CSCs have been demonstrated to play vital roles in carcinogenesis and antitumor therapy. Our previous report found that Krüppel-like family members (KLFs) and zinc finger protein 32 (ZNF32) play oncogenic roles in carcinogenesis. However, the roles and mechanism of ZNF32 in CSCs are still unknown. Our study demonstrated that ZNF32 was highly expressed in colorectal CSCs, which promoted their self-renewal capacity and tumorigenicity. Overexpression of ZNF32 in colorectal cancer (CRC) cells increased their self-renewal capacity. Furthermore, we identified the leptin receptor (LEPR) as the downstream target gene of ZNF32 and verified that the ZNF32-mediated regulation of CRC self-renewal is achieved via the LEPR- signal transducer and activator of transcription 3 (STAT3) pathway. Moreover, ZNF32 regulated the expression of SOX2, a core transcription factor in stem cells. Finally, we demonstrated that ZNF32 and LEPR were positively correlated in CRC tissues. ZNF32 expression was negatively correlated with the prognosis of CRC patients. Therefore, therapeutically targeting the ZNF32-LEPR-STAT3 pathway in the clinic is tempting.

## Introduction

Colorectal cancer (CRC) was the second leading cause of cancer-related death worldwide in 2018, with an incidence rate of 10.2% and a mortality rate of 9.2% [1]. Cancer stem cells (CSCs) were first identified and isolated in acute myeloid leukemia by Lapidot et al. in the early 1990s [2] and represent a subpopulation of cells with distinctive differentiation, proliferation, and self-renewal capabilities in tumor tissues [3, 4]. Subsequently, CSCs have also been identified in other hematological malignancies and a variety of solid tumors [5–11]. Due to their inherent self-renewal characteristics and tumorigenic abilities, CSCs have been demonstrated to play vital roles in tumor development, metastasis, reoccurrence, and resistance to antitumor therapies [3, 12]. In recent years, studies on CSCs have attracted a substantial number of researchers and promoted the understanding of the biological features and regulation of CSCs. Compared to somatic cells, CSCs tend to abnormal overexpress many transcription factors, such as Oct4, Sox2, Nanog, KLF4, and MYC [4, 13]. We and others have also identified various signaling pathways that are involved in the regulation of CSCs, including Wnt, NF-κB, Notch, Hedgehog, JAK-STAT, PI3K/AKT/mTOR, and TGF/SMAD [13].

Krüppel-like factors (KLFs) belong to a group of transcription factors that contain the C-terminus of the zinc finger domain, bind to the target DNA sequence, and are highly conserved in evolution [14]. KLFs not only regulate cell proliferation, differentiation, metabolism, apoptosis, and migration and control physiological processes such as growth, development, and embryogenesis but also regulate the pathogenesis of many diseases, including the onset, development, and maintenance of tumorigenesis [15–17]. KLF4 is closely related to the self-renewal capacity of leukemia stem cells, osteosarcoma stem cells, and colon cancer stem cells [18–21].

Zinc finger protein 32 (ZNF32) is an important transcription factor in the KLFs that has attracted interest from an increasing number of researchers. Previously, we demonstrated that ZNF32 regulates the TGF-β receptor 2 signaling pathway in lung adenocarcinoma to confer multidrug resistance [22]. Notably, we recently showed that ZNF32 can promote breast cancer stem cell-like properties by enhancing GPER transcription [23]. We hypothesized that ZNF32-mediated regulation might also be applicable for other cancer stem cells. In the present study, we sought to investigate the functional roles of ZNF32 and demonstrated that ZNF32 can promote the self-renewal capacity of colorectal CSCs.

## Materials and methods

The details of some experimental procedures were described previously [22], and described in Supplementary Materials and methods.

### Materials and cell culture

Human CRC cell lines SW480, HCT116, and SW620 were purchased from ATCC. These cell lines were authenticated by STR profiling. Primary CRC cells (pCRC1, pCRC2, and pCRC3) were extracted from tumor tissues obtained from the first affiliated hospital of Chengdu medical college. CRC cells were cultured in DMEM (HyClone, USA) with 10% fetal bovine serum (Gibco, Australia) in a 37°C, 5% CO_2_ humidified incubator. CSCs were grown in DMEM / F12 (Hyclone, USA) with EGF (20 ng/μl, Peprotech, USA), β-FGF (20 ng/μl, Peprotech, USA). We screened colorectal CSCs by serum-free suspension culture. AG490 (Sigma) was dissolved in ethanol (5 mg/ml), then used as 20 μM for experiments.

### In vivo experiment

BALB / c nude mice (female, 6-8 weeks) were purchased from Beijing Viton Lihua Experimental Animal Technology Co., Ltd. (Beijing, China). Mice were raised in the specific-pathogen-free facilities of the animal center of Chengdu medical college. CRC cells and colorectal CSCs with different cell numbers (10^3^, 10^5^, 10^7^) were injected subcutaneously to evaluate the tumorigenicity. There are 5 mice in each group. And each group was repeated 3 times independently. All mice were sacrificed 3 weeks after inoculation. All experimental protocols were approved by the animal experimental teaching and research committee of Chengdu medical college, and were performed in accordance with the nation’s relevant laws and animal welfare.

### Flow cytometry (FCM)

According to the manufacturer’s instructions, CD133 antibody was used to detect colorectal CSCs surface markers. Briefly, cells with a density of 1 × 10^6^ / ml were fixed with methanol and incubated with the antibody at 4 °C. After 60 min of incubation, cells were washed 3 times with cold PBS and analyzed using a flow cytometer (BD, USA). By normalizing the fluorescence with the controls, tumor stem cell marker positive cells were identified.

### Immunofluorescence assay (IFA) and Tunel

Cells were fixed with 4% paraformaldehyde for 15 min and then permeabilized with 0.1% Triton X-100 in PBS for 20 min. Cells were blocked with 5% bovine serum albumin (Sigma, Tokyo, Japan) for 60 min. Next, the cells were incubated with CD133 antibody (1: 200) at 4 °C overnight and then with Cy3 labeled secondary antibody for 1 h. The nucleus was stained with DAPI (Biyuntian, China) color core for 10 min. Cells were imaged with an upright fluorescence microscope (Nikon, Japan). Tunel was performed as described previously [22]. And all operations were performed according to the kit instructions (In situ cell death detection kit-POD, 45197300, Roche Group).

### 3D colony-forming assay

The cells were seeded in 96-well plates at 100 cells / well, in which the culture medium was mixed with 50% Matrigel, 50% serum-free DMEM / F12, EGF, and β-FGF. After 14 days of culture, the clonal sphere formation capacity was calculated.

### Limiting dilution assay

Cells were planted into 96-well plates in suspension culture, approximately 1 cell per well. After 14 days of culture in serum-free DMEM / F12 medium supplemented with growth factors EGF and β-FGF, the tumorsphere formation capacity was calculated.

### RNA-sequence analysis

RNA was extracted from CSC-pCRC1, CSC-SW480, pCRC1, and SW480. The RNA-seq analysis was conducted by Yunshen Biological Company, where the downstream target genes of ZNF32 were screened. The RNA-sequence information of CSCs and bulk cells were summarized in Supplementary Table 1.

### Statistical analysis

Statistical analysis was performed with SPSS 21.0 software. Medcalc software is used to calculate the sample size to ensure that there is sufficient capacity to detect the prespecified effect amount. Student’s *t*-test was used for comparison between the two groups, and one-way analysis of variance was used for comparison of multiple groups. In Kaplan–Meier curves survival analysis, log-rank test was used. The variance was similar between the groups that are being statistically compared. *P* < 0.05 was considered statistically significant.

## Results

### Reduced expression of ZNF32 in colorectal CSCs inhibited their self-renewal ability

Colorectal CSCs were enriched by serum-free suspension culture (Fig. S1A). WB and qPCR were performed to detect the expression of stem cell markers CD133, CD166, and ALDH1, the result showed they are most stable and significant highly expressed in CSC-SW480 and CSC-pCRC1 relative to bulk cells (Fig. S1B, C). This was further supported by IFA and FCM (Fig. S1D, E). In addition, the self-renewal capacity and tumorigenicity of CSC-SW480 and CSC-pCRC1 were significantly enhanced compared to bulk cells (Fig. S1F–H). However, in other colorectal CSCs (CSC-SW620, CSC-HCT116, CSC-PC2, and CSC-PCR3), the expression of CD133, CD166, and ALDH1 were not consistent raised relative to bulk cells (Fig. S2A, B). Because CRC is a heterogeneous disease, the status of EGFR, MMR, BRAF, APC, TP53, and KRAS in CRC cells were summarized (Table S1). FCM analysis further demonstrated that CD133 was highly expressed in CSC-SW620 and CSC-pCRC2 (Fig. S2C), and the tumorigenicity of CSC-SW620 and CSC-pCRC2 were significantly enhanced compared to bulk cells (Fig. S2D). The above results indicate that colorectal CSCs screened by serum-free suspension culture have enhanced self-renewal ability compared to bulk cells.

**Fig. 1:**
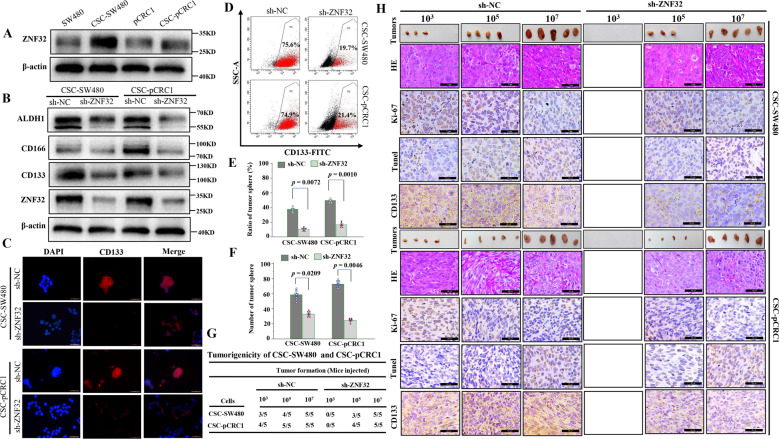
Reduced expression of ZNF32 in colorectal CSCs inhibited their self-renewal ability. (**A**) Western blot detection of ZNF32 expression in CRC cells (SW480 and pCRC1) and colorectal CSCs (CSC-SW480 and CSC-pCRC1). The image is one represent of three independent experiments. (**B**) Western blot analysis of ZNF32, CD133, CD166 and ALDH1 between ZNF32-knockout (sh-ZNF32) and control (sh-NC) in CSC-SW480 and CSC-pCRC1 cells. The image is one represent of three independent experiments. (**C**) IFA to detect CD133, a key marker of colorectal CSC expression, between sh-ZNF32 and sh-NC in CSC-SW480 and CSC-pCRC1 cells. The antigenic determinant of CD133 were located in the membrane, as a result, the positive cells were membranous positive (red). DAPI was used to stain nuclei. The image is one represent of three independent experiments. (micron bar = 50 μm). (**D**) FCM to confirm CD133 expression between sh-ZNF32 and sh-NC in CSC-SW480 and CSC-pCRC1 cells. The image is one represent of three independent experiments. (**E**) 3D colony-forming assay to analyze the colony formation capacity between sh-ZNF32 and sh-NC in CSC-SW480 and CSC-pCRC1 cells. The data presented as the means ± S.Ds. The dots of histogram were used to plot all data. (**F**) Limiting dilution assay to analyze the number of tumor spheres between sh-ZNF32 and sh-NC in CSC-SW480 and CSC-pCRC1 cells. The data presented as the means ± S.Ds. The dots of histogram were used to plot all data. (**G**) CSCs (sh-ZNF32 and sh-NC) with different cell numbers (10^3^, 10^5^, 10^7^) were injected subcutaneously. All mice were sacrificed 3 weeks after inoculation, the tumors were removed, and the tumor formation rate was calculated. There are 5 mice in each group. And each group was repeated 3 times independently. And consistent results were obtained. (**H**). The tumor morphology is shown in the tumor column. And the samples were stained for HE and IHC analysis with Ki-67, CD133 and Tunel. The positive cells were stained brown. The image is one represent of three independent experiments. (micron bar = 20 μm).

And we found that ZNF32 was significantly upregulated in CSC-SW480, CSC-SW620, CSC-pCRC1, and CSC-pCRC2 compared to bulk cells (Figs. 1A and S3B), implying that ZNF32 may play some regulatory roles in colorectal CSCs. To address this hypothesis, stable colorectal CSCs with ZNF32 knockdown were constructed. Our study showed that sh-ZNF32 significantly reduced the expression of ZNF32 and stem cell markers compared to sh-NC group (Figs. 1B and S3C). IFA and FCM also confirmed the remarkable reduction in CD133 expression in sh-ZNF32-transduced colorectal CSCs (Figs. 1C, D and S3A, D). In addition, we found that the colony-forming ability of colorectal CSCs was dramatically impaired by ZNF32 knockdown (Fig. 1E, F). Similarly, an in vivo experiment demonstrated that the tumorigenicity of colorectal CSCs was also significantly compromised by ZNF32 knockdown (Figs. 1G, S3E, and S4A–D). And we further analyzed the cell proliferation (Ki-67) and apoptosis (Tunel) in tumor tissues. After knocking out ZNF32 in colorectal CSCs, the proportion of Ki-67 positive cells were decreased, and the proportion of apoptosis cells was increased (Figs. 1H and S4E, F, H, I), while the CD133-positive cells was also significantly reduced (Figs. 1H and S4G, J). These results suggest that interference with ZNF32 expression reduces the self-renewal capacity of colorectal CSCs.

### Overexpression of ZNF32 in CRC cells increased their self-renewal capacity

**Fig. 2:**
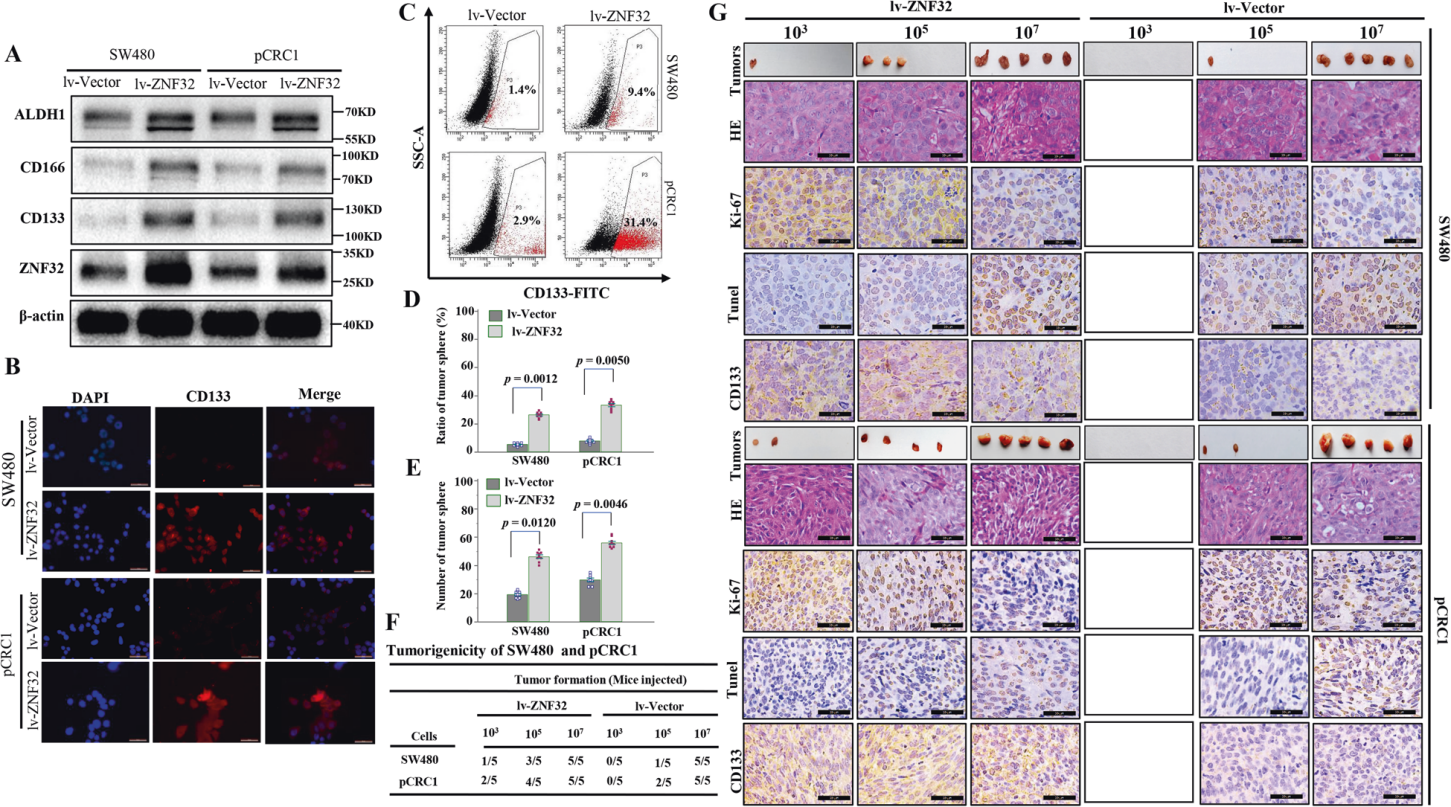
Overexpression of ZNF32 in CRC cells increased the self-renewal capacity. (**A**) Western blot analysis of ZNF32, CD133, CD166 and ALDH1 between ZNF32-overexpressing (lv-ZNF32) and control (lv-Vector) SW480 and pCRC1 cells. The image is one represent of three independent experiments. (**B**) IFA to detect CD133 expression between lv-ZNF32 and lv-Vector in SW480 and pCRC1 cells. The CD133-positive cells were membranous positive (red). The image is one represent of three independent experiments. (micron bar = 50 μm). (**C**) FCM to confirm CD133 expression between lv-ZNF32 and lv-Vector in SW480 and pCRC1 cells. The image is one represent of three independent experiments. (**D**) 3D colony-forming assay to analyze the colony formation capacity between lv-ZNF32 and lv-Vector in SW480 and pCRC1 cells. The data presented as the means ± S.Ds. The dots of histogram were used to plot all data. (**E**) Limiting dilution assay to analyze the number of tumor spheres between lv-ZNF32 and lv-Vector SW480 and pCRC1 cells. The data presented as the means ± S.Ds. The dots of histogram were used to plot all data. (**F**) CRC cells (lv-ZNF32 and lv-Vector) with different cell numbers (10^3^, 10^5^, 10^7^) were injected subcutaneously, and the tumor formation rate was calculated. There are 5 mice in each group. And each group was repeated 3 times independently. (**G**) The tumor morphology is shown in the tumor column. And the samples were stained for HE and IHC analysis with Ki-67, CD133 and Tunel. The positive cells were stained brown. The image is one represent of three independent experiments. (micron bar = 20 μm).

To further verify that ZNF32 expression is associated with the self-renewal capacity of colorectal CSCs, we constructed stable overexpressing ZNF32 CRC cell lines (Fig. 2A). In contrast with the knockdown in colorectal CSCs, overexpression of ZNF32 in CRC cells significantly upregulated the expression of CD133, CD166, and ALDH1 (Fig. 2A). IFA and FCM confirmed that overexpression of ZNF32 in SW480 and pCRC1 cells strikingly increased the proportion of CD133-positive cells, especially in pCRC1 cells (Figs. 2B, C and S3F). In addition, the overexpression of ZNF32 also enhanced the colony-forming capacity and tumorigenicity of SW480 and pCRC1 cells, which showed similar features towards colorectal CSCs (Fig. 2D–F). And tumor growth curve further confirmed that overexpression of ZNF32 can increase the tumorigenicity of SW480 and pCRC1(Fig. S5A–D). And the cell proliferation and apoptosis were further analyzed in tumor tissues. After overexpression of ZNF32 in SW480 and pCRC1 cells, the proportion of Ki-67 positive cells were increased, and the proportion of apoptosis cells was decreased (Figs. 2H and S5E, F, H, I), while the CD133-positive cells was also significantly increased (Figs. 2H and S5G, J). Overall, the above results indicated that overexpression of ZNF32 increased the self-renewal capacity and tumorigenicity of CRC cells.

### RNA-sequence analysis of CRC and colorectal CSCs

**Fig. 3:**
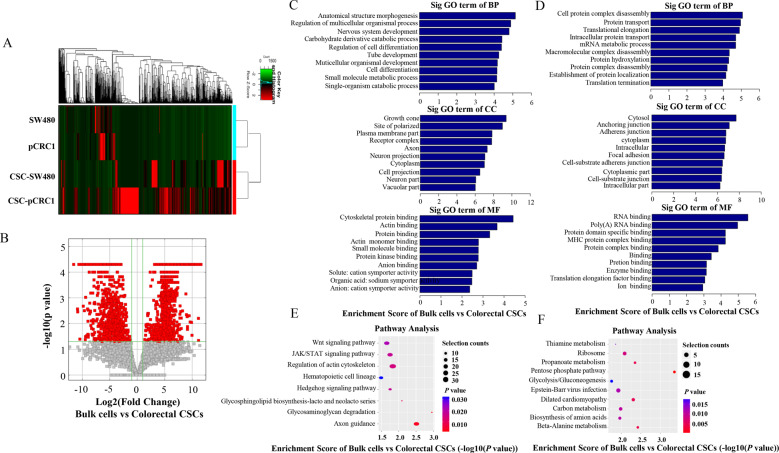
RNA-sequence analysis of CRC cells and colorectal CSCs. (**A**) Heat map of 2718 differentially expressed genes between bulk cells and colorectal CSCs (SW480 and pCRC1 vs CSCs-SW480 and CSC-pCRC1). Each column represents one sample; each row represents one probe set. Red indicates upregulated expression, while green indicates downregulated expression. The image is one represent of three independent experiments. (**B**) A volcano plot was arranged using the fold change and *P* value. The red rectangle represents differentially expressed genes between bulk cells and colorectal CSCs, *P* < 0.05, fold change ≥ 2.0. The image is one represent of three independent experiments. (**C**) GO analysis of upregulated genes between bulk cells and colorectal CSCs. The data presented here are from one representative experiment of three independent experiments. (**D**) GO analysis of downregulated genes between bulk cells and colorectal CSCs. The data presented here are from one representative experiment of three independent experiments. (**E**) KEGG pathway analysis of upregulated genes between bulk cells and colorectal CSCs. The data presented here are from one representative experiment of three independent experiments. (**F**) KEGG pathway analysis of downregulated genes between bulk cells and colorectal CSCs. The data presented here are from one representative experiment of three independent experiments.

Having shown that the expression of ZNF32 is closely related to the self-renewal capacity of colorectal CSCs, we sought to screen the potential downstream genes and signaling pathways involved in ZNF32 regulation. We first performed RNA sequencing to conduct an in-depth comparison analysis of mRNA derived from CSC-pCRC1, CSC-SW480, and their corresponding bulk cells. Any gene whose expression was changed twice or more was selected (Table S2). This revealed 1,818 and 900 genes that were significantly upregulated and downregulated, respectively (Fig. 3A, B). Next, we conducted gene ontology (GO) term enrichment analysis. There was considerable alteration in terms of a variety of biological process (BP), cellular component (CC), molecular function (MF) (Fig. 3C, D). In particular, activated genes were majorly related to cell differentiation. We also performed the Pathway (KEGG) Analysis (Fig. 3E, F), which identified that JAK/STAT signaling pathway was significantly activated in colorectal CSCs.

### ChIP-sequence analysis of CRC cells

**Fig. 4:**
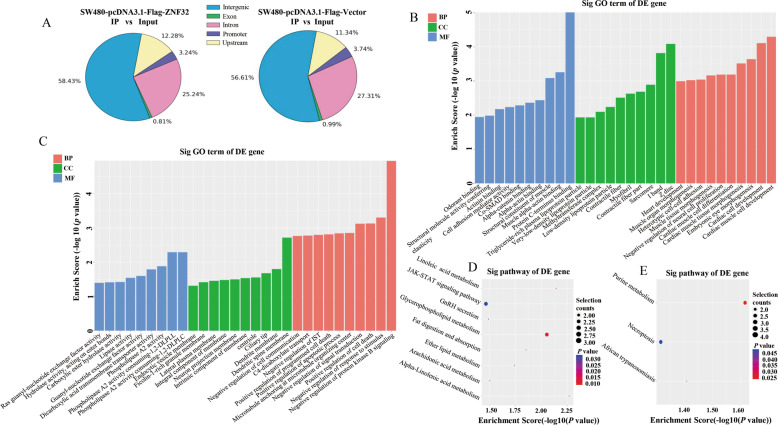
ChIP-sequence analysis of CRC cells. (**A**) ChIP-sequence analysis identified 1,235 peak genes in the SW480-pcDNA3.1-Flag-ZNF32 group and 1639 peak genes in the SW480-pcDNA3.1-Flag-Vector group. The data presented here are from one representative experiment of three independent experiments. (**B**) GO analysis of upregulated genes between SW480-pcDNA3.1-Flag-ZNF32 and SW480-pcDNA3.1-Flag-Vector cells. The data presented here are from one representative experiment of three independent experiments. (**C**) GO analysis of downregulated genes. The data presented here are from one representative experiment of three independent experiments. (**D**) KEGG pathway analysis of upregulated genes between SW480-pcDNA3.1-Flag-ZNF32 and SW480-pcDNA3.1-Flag-Vector cells. The data presented here are from one representative experiment of three independent experiments. (**E**) KEGG pathway analysis of downregulated genes between SW480-pcDNA3.1-Flag-ZNF32 and SW480-pcDNA3.1-Flag-Vector cells. The data presented here are from one representative experiment of three independent experiments.

Next, we conducted ChIP-sequence analysis to further identify the downstream genes regulated by ZNF32 in CRC cells. SW480 cells were transfected with the plasmid expressing FLAG-tagged ZNF32, with the FLAG-tagged empty vector as a control. The results showed that SW480-pcDNA3.1-Flag-ZNF32 identified 1,235 peak genes, 58.43% of which were located in the intergenic region and 3.24% at the promoter region (Fig. 4A). More peak genes (1,639) were identified in the SW480-pcDNA3.1-Flag-Vector group, with 56.61% in the intergenic region and 3.74% in the promoter area (Fig. 4A). Differentially enriched regions of the promoter for SW480-pcDNA3.1-Flag-ZNF32 compared to the SW480-pcDNA3.1-Flag-Vector group are summarized in Table S3. Further GO analysis revealed the functional changes of differential genes in BP, CC, and MF (Fig. 4B, C). The further signaling pathways analysis demonstrated that glycerophospholipid metabolism and JAK-STAT signaling pathway were activated (Fig. 4D), while purine metabolism and necroptosis were inhibited (Fig. 4E).

### ZNF32 regulated the self-renewal capacity of colorectal CSCs through the LEPR-STAT3 signaling pathway

**Fig. 5:**
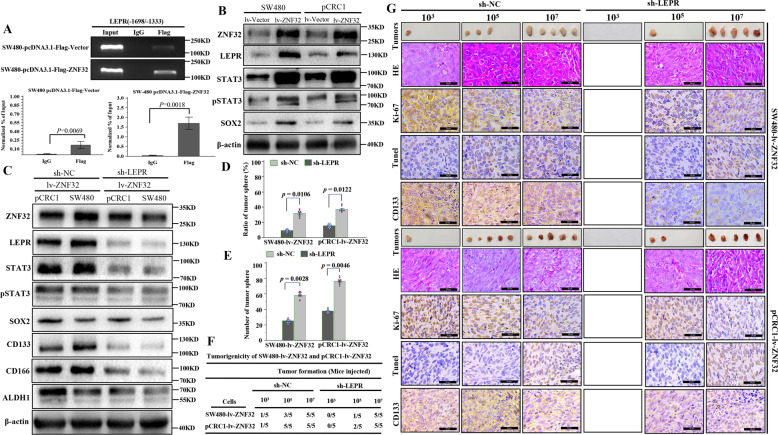
ZNF32 regulated the self-renewal capacity of colorectal CSCs through the LEPR-STAT3 signaling pathway. (**A**) DNA fragments from SW480-pcDNA3.1-Flag-ZNF32 and SW480-pcDNA3.1-Flag-Vector cells were immunoprecipitated with Flag-specific antibodies and analyzed via RT-PCR using the indicated LEPR (-1698/-1333) primers. The data presented here are from one representative experiment of three independent experiments and are presented as the means ± S.Ds. (**B**) Western blot analysis of ZNF3, LEPR, STAT3, pSTAT3 and SOX2 between lv-ZNF32 and lv-Vector SW480 and pCRC1 cells. SW480 and pCRC1 cells with overexpression of ZNF32 and knockdown of LEPR, Western blot analysis to detect the expression of LEPR, SOX2, STAT3 and pSTAT3 and the stem cell markers CD133, CD166 and ALDH (**C**), the image is one represent of three independent experiments. 3D colony-forming assay to analyze the colony formation capacity. The data presented as the means ± S.Ds. The dots of histogram were used to plot all data (**D**), and limiting dilution assay to analyze the number of tumor spheres. The data presented as the means ± S.Ds. The dots of histogram were used to plot all data (**E**). In vivo experiments to confirm the tumorigenicity of sh-LEPR and sh-NC between SW480-lv-ZNF32 and pCRC1-lv-ZNF32 cells. There are 5 mice in each group. Each experiment was performed at least in triplicate, and consistent results were obtained (**F**). (**G**). The tumor morphology is shown in the tumor column. And the samples were stained for HE and IHC analysis with Ki-67, CD133 and Tunel. The positive cells were stained brown. The image is one represent of three independent experiments. (micron bar = 20 μm).

Based on the analysis of RNA sequencing and ChIP-sequence analysis, the JAK-STAT signaling pathway was screened by both methods, suggesting that it plays an important role of self-renewal in colorectal CSCs. We further analyzed the JAK-STAT signaling pathway-associated genes changes, and found that IL19//LEPR//PIAS4 is the most significant upregulated genes (Table S4). Previously, we confirmed GA/CATTT as the transcriptional binding site of ZNF32 [23]. By analyzing the promoters of IL19//LEPR//PIAS4, we found that the promoter region of leptin receptor (LEPR) contained two transcriptional binding sites of ZNF32 (Fig. S6A). ChIP analysis further confirmed that ZNF32 binds the promoter region of LEPR (Fig. 5A), indicating that LEPR was the downstream target gene of ZNF32. To further verify the role of LEPR in CRC, we analyzed the expression of LEPR and its downstream signal STAT3 in colorectal CSCs. We found that in CSC-SW480 and CSC-pCRC1 cells, LEPR expression was increased, as well as downstream STAT3 and activated pSTAT3 (Fig. S6B, C). In addition, we detected the expression of another transcription factor, SOX2, which was also significantly elevated in colorectal CSCs (Supplementary Fig. 6B). Similarly, in SW480 and pCRC1 cells with ZNF32 overexpressed, the expression of LEPR, STAT3, pSTAT3, and SOX2 was significantly upregulated compared to that in the control cells (Fig. 5B). To further verify the relationship between ZNF32 and LEPR and their role in colorectal CSCs, stable SW480 and pCRC1 cells overexpressing ZNF32 and with knockdown of LEPR were constructed. We found that the expression of LEPR, STAT3, and pSTAT3 was significantly downregulated (Fig. 5C), as were CD133, CD166, and ALDH. Notably, SOX2 expression also decreased, but to a lesser extent (Fig. 5C). In addition, in order to prove the axis involved in stem cell regulation, we used AG490, a specific inhibitor of STAT3 phosphorylation, to further analyze the regulatory effect of ZNF32 on STAT3 signaling pathway (Fig. S6D). And interfering with LEPR expression significantly inhibited CRC cells colon formation and tumor formation (Figs. 5D–F and 7A–D). Furthermore, we confirmed that knocked out the LEPR gene in SW480-lv-ZNF32 and pCRC1-lv-ZNF32, the CD133 and Ki-67 positive cells were decreased, and the proportion of apoptosis cells was increased in tumor tissues (Figs. 5G and S7E–J). Collectively, the above results indicate that ZNF32 regulated the self-renewal capacity and tumorigenicity of colorectal CSCs through the LEPR-STAT3 signaling pathway.

### ZNF32-LEPR signaling was negatively correlated with the survival of CRC patients

**Fig. 6:**
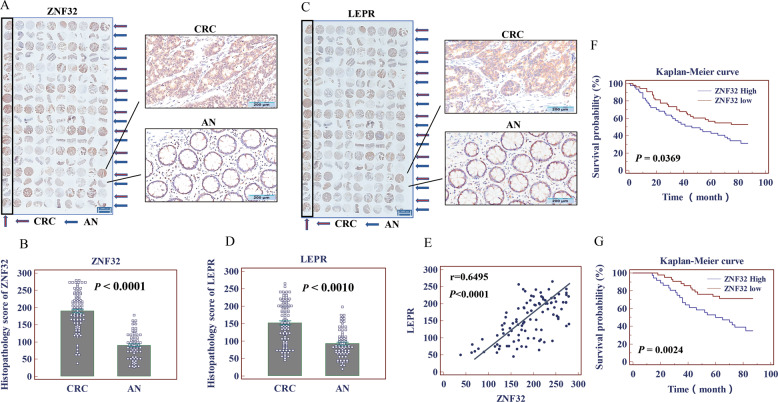
The ZNF32-LEPR signal was negatively correlated with the survival of CRC patients. (**A**, **B**) IHC was used to analyze the expression of ZNF32 in tumor tissue (CRC) and adjacent normal tissue (AN). The positive cells were stained brown. The image is one represent of three independent experiments. (micron bar = 200 μm). (**C**, **D**) IHC to analyze the expression of LEPR in CRC and AN. The positive cells were stained brown. The image is one represent of three independent experiments. (micron bar = 50 μm). (**E**) Correlation analysis demonstrated that ZNF32 expression was positively correlated with LEPR expression in CRC tissues. (**F**) The survival status of patents was followed up, and Kaplan–Meier survival curves for these 100 patients were categorized into two groups based on the ZNF32 IHC score. (**G**) Kaplan–Meier survival curve for 78 patients (36 with high and 42 with low ZNF32 expression) who had received different cycles of chemotherapy (platinum combined with fluorouracil). Each experiment was performed at least in triplicate, and consistent results were obtained. The data presented here are from one representative experiment of three independent experiments and are presented as the means ± S.Ds.

Finally, we investigated ZNF32 and LEPR expression in clinical tumor specimens from CRC patients. We collected tumor specimens from 100 patients, 80 of whom had both tumor tissue (CRC) and normal tissue adjacent (AN) to the tumor. The clinicopathological factors of CRC patients are summarized in Table 1. First, our data showed that the expression of ZNF32 in CRC was significantly higher than that in AN (Fig. 6A, B) (*p* < 0.0001). Similarly, the expression of LEPR in tumor tissue was also remarkably elevated compared with that in normal tissues (Fig. 6C, D) (*p* < 0.001). But very interesting, by analyzing the public dataset GEPIA (http://gepia.cancerpku.cn/), we found that there was no significant difference in the expression of ZNF32 and LEPR in CRC tumor tissue compared to healthy mucosa (Fig. S8A, C). Correlation analysis of the histopathological scores of ZNF32 and LEPR demonstrated that the expression of the two genes was positively correlated in CRC tissues (*r* = 0.6495, *p* < 0.0001, Fig. 6E). Next, based on the histopathological score of ZNF32, we set up the average value to define high or low expression. Of the 100 specimens, 47 were defined as high and 53 as low expression of ZNF32. It is worth noting that ZNF32 and LEPR expression showed strikingly positive associations with the TNM stage of patients (*p* = 0.0042 and 0.0193, respectively) but not with other parameters (Table 1). In addition, the survival status of these 100 patients was followed up and reviewed from January 2010 to December 2018, which showed that patients with high ZNF32 expression generally had a shorter survival (*p* = 0.0369, Fig. 6F). Furthermore, of the 100 patients, 78 (36 with high and 42 with low ZNF32 expression) had received different cycles of chemotherapy (platinum combined with fluorouracil). Notably, further survival analysis found that among the 78 patients, patients with high ZNF32 expression had a remarkably shorter survival (*p* = 0.0024, Fig. 6G). We further used TCGA data (https://www.cancer.gov/tcga) to analysis the prognosis information of ZNF32 and LEPR in CRC patients. The results showed that the expression of ZNF32 was negatively correlated with the prognosis of CRC patients, which was consistent with our research (Fig. S8B). And the expression of LEPR is negatively correlated with the prognosis in CRC patients before 100 months (Fig. S8C). Therefore, our results demonstrated that both ZNF32 and LEPR were highly expressed in CRC tissues, and were negatively correlated with the prognosis of CRC patients.

**Table 1:** The clinicopathological factors of CRC patients.

Characteristics		Cases	ZNF32		*P* Value	LEPR		*P* Value
			High	Low		High	Low	
Gender	Male	58	28	30	0.9224	31	27	0.8706
	Female	42	19	23		24	18	
Age(years)	≤60	25	10	15	0.5630	15	10	0.7277
	>50	75	37	38		40	35	
Liver metastasis	Yes	5	4	1	0.2904	3	2	0.8176
	No	95	43	52		52	43	
TNM stage	I and II	46	14	32	0.0042*	19	27	0.0193*
	III and IV	54	33	21		36	18	
Differentiation stage	Well	17	6	11	0.0754	9	8	0.1288
	Moderate	59	25	34		27	22	
	Poor	24	16	8		19	5

## Discussion

Because CSCs comprise only a small fraction of heterogeneous tumor cell populations (<1% in solid tumors), their enrichment is generally challenging [24]. In the present study, we employed a serum-free suspension culture method to enrich colorectal CSCs from the CRC cell lines and CRC primary cells. A group of cell surface markers are generally used to define colorectal CSCs, including CD44, CD133, CD166, CD24, EpCAM, LGR5, and ALDH [25, 26]. The expression of CD133 is one of the most important features of colorectal CSCs. Isolated single CD133^+^ CRC cells show self-renewal and multi-lineage differentiation [27]. And more study demonstrated that CD166 was an additional differentially expressed marker for colorectal CSCs [25]. In addition, High expression of ALDH1 has been identified as a CSC marker in various types of cancer, including CRC [25]. So, we detected the expression of CD133, CD166, and ALDH. And our data showed significantly higher expression of these markers in the enriched CSC-SW40 and CSC-pCRC1, indicating the high efficiency of enrichment. However, in the other colorectal CSCs, the expression of CD133, CD166, and ALDH1 were not consistent raised relative to bulk cells. This indicates that these stem markers were expressed differently in different colorectal CSCs. Because CRC is a heterogeneous disease, KRAS activates, microsatellite instability and mutation of APC are all related to the phenotype of colorectal CSCs [28–30]. And the status of EGFR, MMR, BRAF, APC, TP53 in CRC cells were different. So, the differential expression of CSC markers in different colorectal CSCs may related to the mutation of these genes.

ZNF32 has been shown to be expressed in many cancer cells, including CRC cells. In our present study, we revealed that compared to their bulk cells, colorectal CSCs had remarkably elevated ZNF32 expression, suggesting that ZNF32 might play an important role in colorectal CSCs. Through two independent experiments with different strategies, we demonstrated that the expression of ZNF32 in CRC cells was closely related to differentiation towards colorectal CSCs, which was positively correlated with the expression of the colorectal CSC markers CD133, CD166, and ALDH1. Notably, we further showed that ZNF32 expression was also well associated with the self-renewal capacity of these colorectal CSCs. Similarly, another research group recently found that ZNF207 is required for the self-renewal and pluripotency of human embryonic stem cells [31]. Jen et al. also demonstrated that ZNF322A promotes lung tumorigenesis as a transcription suppressor of c-Myc expression [32]. We previously demonstrated that in lung adenocarcinoma, ZNF32 contributes to the induction of multidrug resistance [22]. Our results in the present study provide another possible explanation that the high expression of ZNF32 in cancer cells facilitates their differentiation toward CSCs, which are known to be drug resistant [33, 34]. In fact, our analysis of clinical specimens in this study showed that patients with higher ZNF32 expression generally have a shorter survival than those with lower ZNF32 expression, despite the multiple cycles of treatment with platinum-based combined fluorouracil-based chemotherapy they previously received.

The JAK-STAT3 pathway is a primary signaling pathway and plays a crucial role in many cellular processes [35]. It has also been reported that aberrant activation of this pathway is associated with many cancers [36, 37]. And it has been reported that persistent activation of STAT3, and the phosphorylation level of STAT3 may be associated with the poor prognosis of cancer [37]. Intriguingly, our RNA-seq and ChIP-seq results both indicated that genes from the JAK-STAT pathway were upregulated. Based on our previous report that the transcriptional binding site of ZNF32 is GA/CATTT [23], we further screened the key genes in the JAK/STAT pathway and identified the LEPR gene as the downstream target of ZNF32. Leptin is a hormone and inflammatory cytokine that is involved in the regulation of appetite, metabolism and angiogenesis [38]. Our previous study demonstrated that hyperleptinemia directly affects testicular structure and function through the SOCS3/pSTAT3 pathway [39]. Through binding to the receptor LEPR, leptin/LEPR signaling has been shown to be related to the progression of a large number of cancers, such as pancreas and colon cancers [38]. In particular, accumulating evidence has demonstrated that leptin/LEPR binding strongly influences the activation of the JAK/STAT pathway through the phosphorylation of JAK2 and STAT3 [40]. This is in accordance with our results that knockdown of LEPR significantly downregulated the expression of both STAT3 and pSTAT3. We also showed that the silenced expression of LEPR remarkably compromised the expression of CSCs markers, colony-forming ability, and tumorigenicity. Similar to our findings, Zheng et al. showed that LEPR is required for self-renewal capacity of triple-negative breast cancer [41]. Notably, despite the important regulatory role of the LEPR-STAT3 pathway in CSCs, limited upstream regulators of STAT3 have been identified. Recently, one research group first revealed that HN1L can promote breast CSCs properties through the LEPR-STAT3 pathway [42]. We also found that SOX2 was positively associated with ZNF32 in both CRC cells and colorectal CSCs. Our previous work demonstrated that ZNF32 regulates SOX2 expression in zebrafish nervous lateral line system regeneration [43]. However, the mechanism by which ZNF32 regulates SOX2 in colorectal CSCs needs to be further studied. Otherwise, CD133, CD166, and ALDH1 are surface markers of colorectal CSCs. In addition to us, many reports confirming the association of STAT3 signaling pathways with the expression of CD133, CD166, and ALDH1 in CSCS. However, there is no definite evidence that STAT3 directly regulates the expressions of CD133, CD166, and ALDH1. And how STAT3 involved in the regulation of these markers still unknow. In addition, our analysis of clinical specimens revealed that both ZNF32 and LEPR have a high level of positively correlated expression in CRC tissues compared to adjacent normal tissues. Patients with higher levels of ZNF32 expression tend to have shorter survival than those with lower ZNF32 expression.

In conclusion, our study demonstrates that ZNF32 is highly expressed in colorectal CSCs, promotes the self-renewal capacity of colorectal CSCs and is associated with the clinical prognosis of CRC patients. Importantly, we identified LEPR as the downstream target gene of ZNF32 and verified that ZNF32-mediated regulation occurs through the LEPR-STAT3 pathway. It will be tempting to therapeutically target this pathway in the clinic. In fact, an LEPR antagonist was recently tested in a preclinical murine model of triple-negative breast cancer and showed improved survival benefits [44]. More studies involving ZNF32 and LERP will enhance the clinical significance of the ZNF32-mediated LEPR-STAT3 pathway.

## Supplementary information

Supplementary materials

Supplementary figure 1

Supplementary figure 2

Supplementary figure 3

Supplementary figure 4

Supplementary figure 5

Supplementary figure 6

Supplementary figure 7

Supplementary figure 8

Supplementary table 1

Supplementary table 2

Supplementary table 3

Supplementary table 4

Author changes

## Data Availability

RNA sequencing to conduct an in-depth comparison analysis of mRNA derived from CSC-pCRC1, CSC-SW480 and their corresponding bulk cells are included in Supplementary Table 2. ChIP-sequence analysis of the differentially enriched regions of the promoter for SW480-pcDNA3.1-Flag-ZNF32 compared to the SW480-pcDNA3.1-Flag-Vector group are summarized in Supplementary Table 3.
