# Supplementary material for: ZNF32 promotes the self-renewal of colorectal cancer cells by regulating the LEPR-STAT3 signaling pathway
Source: Cell Death Dis. 2022 Feb 3;13(2):108. doi: 10.1038/s41419-022-04530-4 (PMC8814143; doi:10.1038/s41419-022-04530-4)
Supplement: Supplementary file 1 — Supplementary materials [file 41419_2022_4530_MOESM1_ESM.docx]

**Supplementary Materials and methods**

**Quantitative real-time PCR (qPCR)**

The details of the experimental procedures were described previously ^22^. The following gene-specific primers were used: ZNF32 forward, 5′-AGAATGTAGCGTT CTTCAATGTG-3′, and reverse, 5ʹ-CCTGTA GTGTCTTCGAATCTGG-3ʹ; ALDH1 forward, 5ʹ- CTCCTCTCACGGC TCTTCAC-3′, and reverse, 5ʹ- CCATGGTGTGC AAACTCAAC-3ʹ; CD133 forward, 5ʹ- AACAGCACCTTGAAGAGCTT-3ʹ, and reverse, 5ʹ-TCCATCAAGTGAAACCTGCA-3ʹ; CD166 forward, 5ʹ-TATTGCCTT CAGATCCTCTA-3ʹ, and reverse, 5ʹ-AGTCAAGGTG TTCAGTAAGT-3ʹ; STAT3 forward, 5ʹ-AATATCCTCAGTTATCCCT-3ʹ, and reverse, 5ʹ-TGTTGGAGACCAG AGTTTG-3ʹ; LEPR forward, 5ʹTGTTCTACTCAGACTCATA-3ʹ, and reverse, 5ʹ-CTAGAAATAAGCCCAACAG-3ʹ; and β-actin forward, 5ʹ-AAGGTGACAGCAGT CGGTTGG-3ʹ, and reverse, 5ʹ-GGCAAGGGACTTCCTGTAACA ATG-3ʹ.

**Western blot analysis (WB)**

The details of the experimental procedures were described previously ^22^. The antibodies used as follows: The ZNF32 monoclonal antibody was made by our research group ^24^, CD133 (EPR20980-104, ab216323), CD166 (EPR2759 (2), ab109215), ALDH1(EP1933Y, ab52492) and LEPR (EPR10193 (B), ab154799) from Abcam, USA; STAT3(124H6, 9139), pSTAT3 (D3A7, 4093), SOX2(D6D9, 3579), and β-actin (8H10D10, 3700) from Cell Signaling Technology, USA. The dilution factor of all the antibodies was 1: 1000 except for β-actin (1: 3000). Anti-rabbit IgG (1: 3000, 14708) and anti-mouse IgG (1: 3000, 7076) from Cell Signaling Technology, USA were used as secondary antibodies.

**Hematoxylin and eosin staining (HE) and immunohistochemistry (IHC)**

Tumors from BALB/c nude mice were fixed in formalin, embedded in paraffin and sliced. HE and IHC was performed as described previously^22^. The following antibodies were used: mouse anti-Ki-67 antibody (1:50 dilution; ab16667, Abcam, USA), mouse anti-CD133 antibody (1:50 dilution; 250142, ZEN BIO China), and anti-mouse immunoglobulin (IgG) (1:800 dilution; Zhongshan, Beijing, China). CRC tissues samples were collected from the First Affiliated Hospital of Chengdu Medical College. Prior written and informed consent was obtained from each patient, and the study was approved by the Ethics Committee of the first affiliated hospital of Chengdu medical college. Our study was performed in accordance with the Declaration of Helsinki. And we used image J software (NIH Image, Bethesda, MD) to calculate the histopathology score in each group. The ki-67 positive expressed in cell nucleus, and the CD133 positive expressed in cell membrane. The expression of ki-67 and CD133 was scored by proportion and intensity. The proportion score represented the estimated proportion of tumor cells staining positive from 0-100. Any brown nuclear staining in cancer cells was counted toward the proportion score. The intensity score represented the average intensity of the positive cells was as follows: 1 (none), 2 (weak), 3 (intermediate) and 4 (strong). The total score was proportion multiplied by intensity scores.

**Immunoprecipitation assays (ChIP)**

SW-480 cells were transfected the ZNF32 expression plasmid pcDNA3.1 flag-ZNF32. The ChIP assay was conducted with Millipore Magna CHIP^TM^ A (Catalog # 17-610) EZ-Magna kit, following the operation procedure. Briefly, cells were cross-linked with 1% formaldehyde at room temperature for 15 minutes. Formaldehyde was quenched with 125mM glycine for 5 minutes, and the cells were collected and washed. Cells and nuclei were lysed sequentially, followed by incubation with anti-Flag antibody (CST, United Kindom) at 4 ° C overnight. The bound DNA was analyzed by qPCR. Sequencing and data analysis were completed by Kang Cheng Biological Company. The ChIP information was summarized in Supplementary Table 2. Primers used for PCR analysis were: LEPR gene promoter, -1698 TAAACTGAGCAAGGATC ATGAAAC-1674 (forward); -1359TAAGTACA CAATGGCTCTCAGAGATC-1333 (reverse).

**Supplementary figures legend**

**Supplementary figure 1. (A).** Serum-free suspension culture to screen cancer stem cells from colon cancer cell line SW480 and primary colon cancer cell line pCRC1. **(B).** PCR to detect the stem cell markers CD133, CD166, ALDH1 expression in colorectal CSCs and bulk cells (CSC-SW480 and CSC-pCRC1 vs SW480 and pCRC1). (**C**). Western Blot to detect the stem cell markers CD133, CD166, ALDH1 expression in enriched colorectal CSCs and bulk cells. **(D).** IFA to detect the CD133 expression in CSC-SW480 and CSC-pCRC1 cells compared to SW480 and pCRC1. **(E).** FCM to confirm CD133 expression in CSC-SW480 and CSC-pCRC1 cells compared to SW480 and pCRC1. (**F**). 3D colony-forming assay to analyze the colon formation capacity in enriched CSC-SW480 and CSC-pCRC1 cells compared to SW480 and pCRC1. Data presented here is from one representative experiment of three independent experiments, and is presented as means ± S.D. (**G**). Limiting dilution assay to analyze the number of tumor sphere in enriched CSC-SW480 and CSC-pCRC1 cells compared to SW480 and pCRC1. Data presented here is from one representative experiment of three independent experiments, and is presented as means ± S.D. (**H**). CSCs cells and bulk cells with different cell numbers (10^3^, 10^5^, 10^7^) were injected subcutaneously, and the tumor formation rate was calculated. Each experiment was performed at least in triplicate, and consistent results were obtained.

**Supplementary figure 2. (A).** PCR to detect CD133, CD166, ALDH1 expression in colorectal CSCs and bulk cells (CSC-SW620, CSC-HCT116, CSC-pCRC2 and CSC-pCRC3 vs SW620, HCT116, pCRC2 and pCRC3). (**B**). Western Blot to detect the stem cell markers CD133, CD166, ALDH1 expression in enriched colorectal CSCs and bulk cells. **(C).** FCM to confirm CD133 expression in CSC-SW620 and CSC-pCRC2 cells compared to SW480 and pCRC1. (**D**). CSCs cells and bulk cells with different cell numbers (10^3^, 10^5^, 10^7^) were injected subcutaneously, and the tumor formation rate was calculated. Each experiment was performed at least in triplicate, and consistent results were obtained.

**Supplementary figure 3. (A).** Statistical analysis of three independent experiments of FCM to confirm CD133 expression between sh-ZNF32 and sh-NC in CSC-SW480 and CSC-pCRC1 cells. The dots of histogram were used to plot all data. **(B, C).** Western blot analysis of ZNF32, CD133, CD166 and ALDH1 between ZNF32-knockout (sh-ZNF32) and control (sh-NC) in CSC-SW620 and CSC-pCRC2 cells. The image is one represent of three independent experiments. **(D).** FCM to confirm CD133 expression between sh-ZNF32 and sh-NC in CSC-SW620 and CSC-pCRC2 cells. The image is one represent of three independent experiments. (**E**). CSCs cells and bulk cells with different cell numbers (10^3^, 10^5^, 10^7^) were injected subcutaneously, and the tumor formation rate was calculated. Each experiment was performed at least in triplicate, and consistent results were obtained. **(F).** Statistical analysis of three independent experiments of FCM to confirm CD133 expression between lv-ZNF32 and lv-Vector in SW620 and pCRC2 cells. The dots of histogram were used to plot all data.

**Supplementary figure 4.** The tumor growth curve of CSC-SW480 and CSC-pCRC1(A-D). Image J software to calculate the histopathology score in CSC-SW480 and CSC-pCRC1. (E, H). Histopathology score of Ki-67 in tumor tissues. (F, I). The percent of Tunle positive cell in tumor tissues. (G, J). Histopathology score of CD133 in tumor tissues. Data presented here is from one representative experiment of three independent experiments, and is presented as means ± S.D. *, *P*<0.05. ^#^, No significant.

**Supplementary figure 5.** The tumor growth curve of SW480 and pCRC1(A-D). Image J software to calculate the histopathology score in SW480 and pCRC1. (E, H). Histopathology score of Ki-67 in tumor tissues. (F, I). The percent of Tunle positive cell in tumor tissues. (G, J). Histopathology score of CD133 in tumor tissues. Data presented here is from one representative experiment of three independent experiments, and is presented as means ± S.D. *, *P*<0.05. ^#^, No significant.

**Supplementary figure 6. (A).** Schematic representation of the ZNF32-binding sites in the TGF-βR2 promoter, and the transcription start site is indicated by +1. **(B).** PCR to detect LEPR, STAT3 expression in colorectal CSCs and bulk cells. **(C).**Western Blot to detect ZNF32, LEPR, STAT3, pSTAT3 and SOX2 expression in CSC-SW480 and CSC-pCRC1 compared to SW480 and pCRC1. **(D).** SW480 and pCRC1 cells with overexpression of ZNF32 and treated by AG490, Western blot analysis to detect the expression of LEPR, SOX2, STAT3 and pSTAT3 and the stem cell markers CD133, CD166 and ALDH. Each experiment was performed at least in triplicate, and consistent results were obtained. Data presented here is from one representative experiment of three independent experiments, and is presented as means ± S.D.

**Supplementary figure 7.** The tumor growth curve of SW480-lv-ZNF32 and pCRC1-lv-ZNF32. (A-D). Image J software to calculate the histopathology score in SW480-lv-ZNF32 and pCRC1-lv-ZNF32. (E, H). Histopathology score of Ki-67 in tumor tissues. (F, I). The percent of Tunle positive cell in tumor tissues. (G, J). Histopathology score of CD133 in tumor tissues. Data presented here is from one representative experiment of three independent experiments, and is presented as means ± S.D. *, *P*<0.05. ^#^, No significant.

**Supplementary figure 8.** In the GEPIA, we have analyzed the expression of ZNF32 for comparison of tumor tissue and healthy mucosa **(A)**, while the expression of LEPR in healthy mucosa and CRC tumor tissue (**C**). TCGA data used to analyze the prognosis information of ZNF32 and LEPR in colon cancer patients (B, D). All of these data were cited from GEPIA (http://gepia.cancer-pku.cn/).
