# Supplementary figures and images for: ZNF32 promotes the self-renewal of colorectal cancer cells by regulating the LEPR-STAT3 signaling pathway
Source: Cell Death Dis. 2022 Feb 3;13(2):108. doi: 10.1038/s41419-022-04530-4 (PMC8814143; doi:10.1038/s41419-022-04530-4)

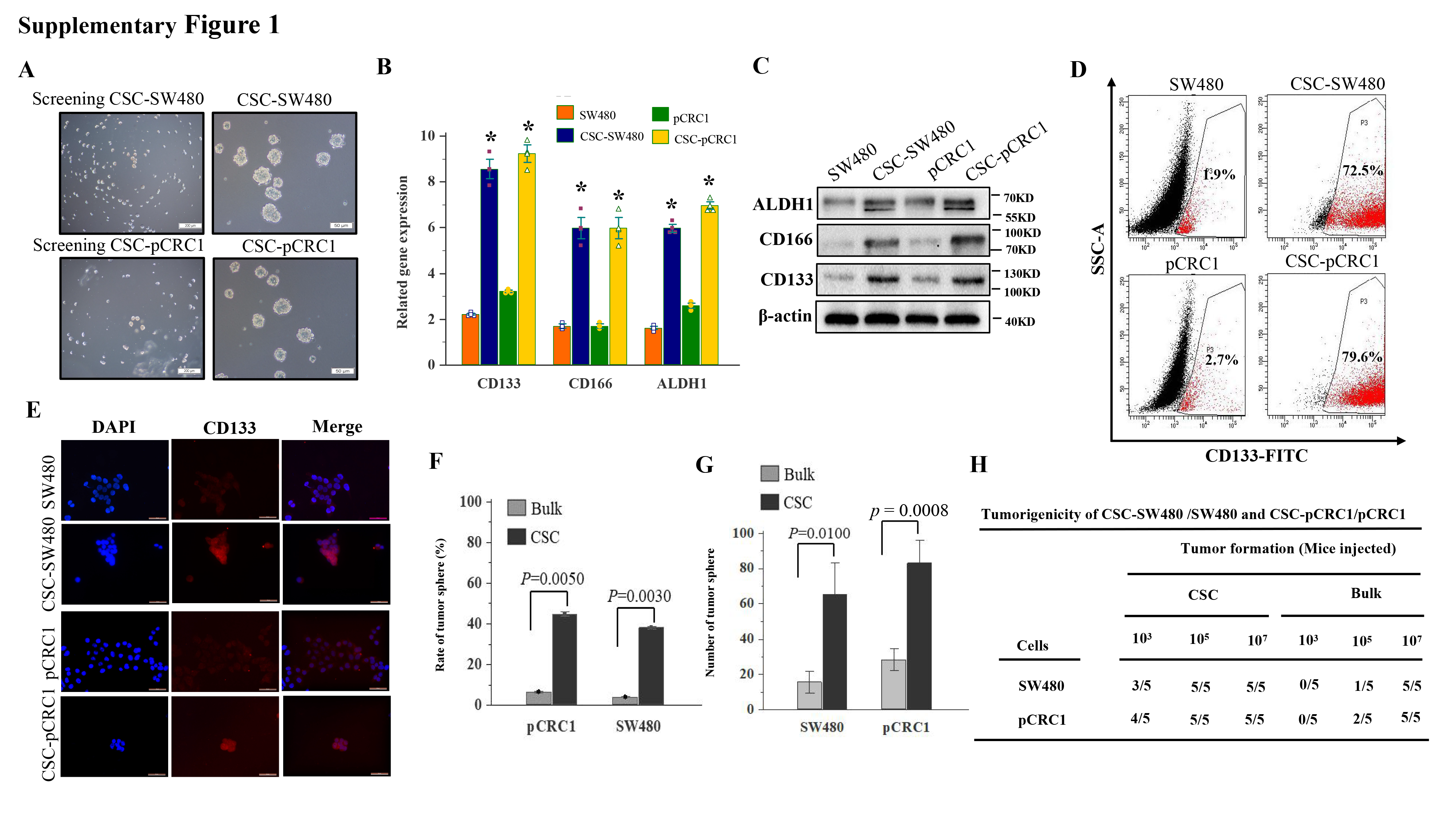

Supplement: Supplementary file 2 — Supplementary figure 1 [file 41419_2022_4530_MOESM2_ESM.tif]

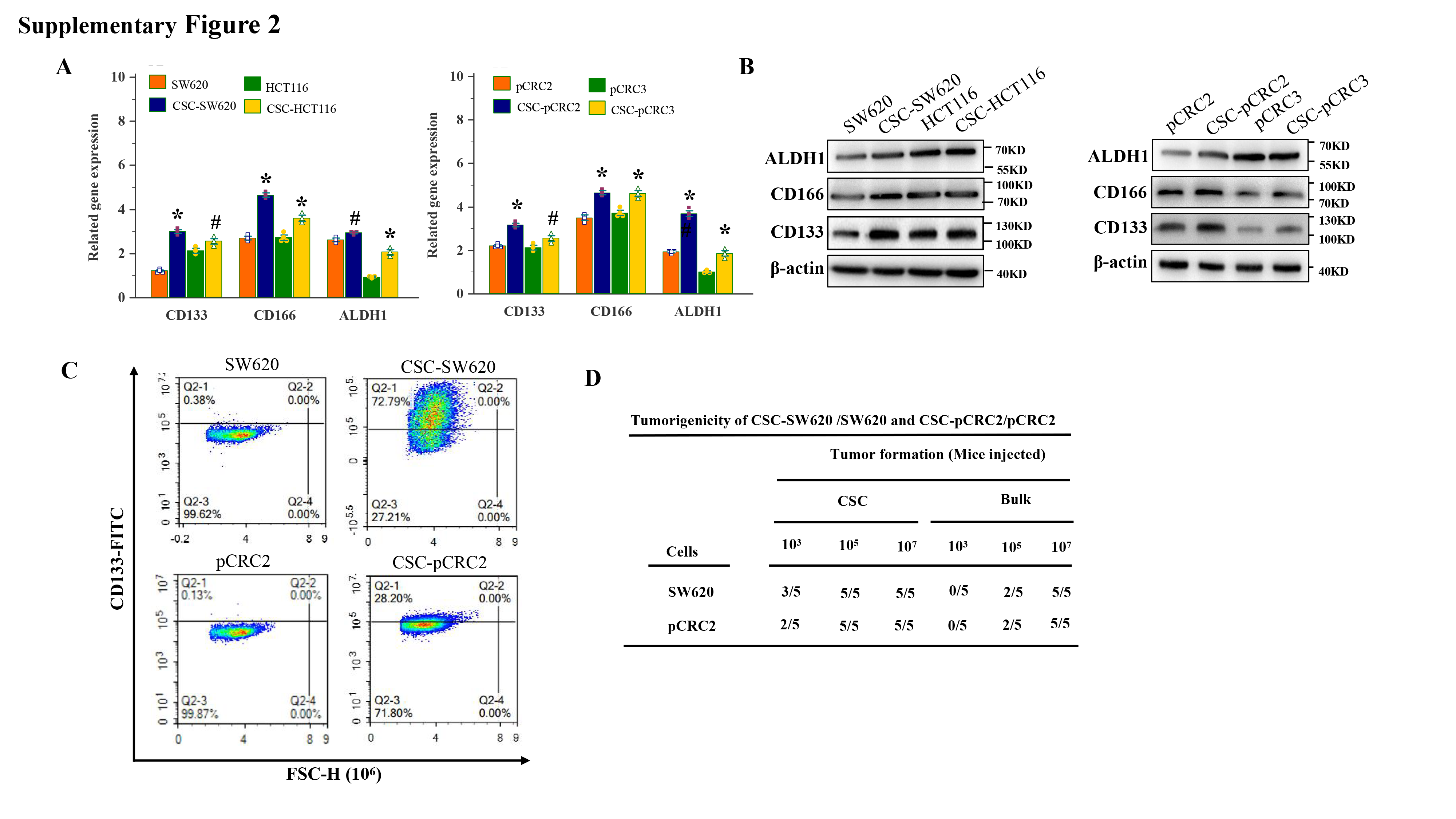

Supplement: Supplementary file 3 — Supplementary figure 2 [file 41419_2022_4530_MOESM3_ESM.tif]

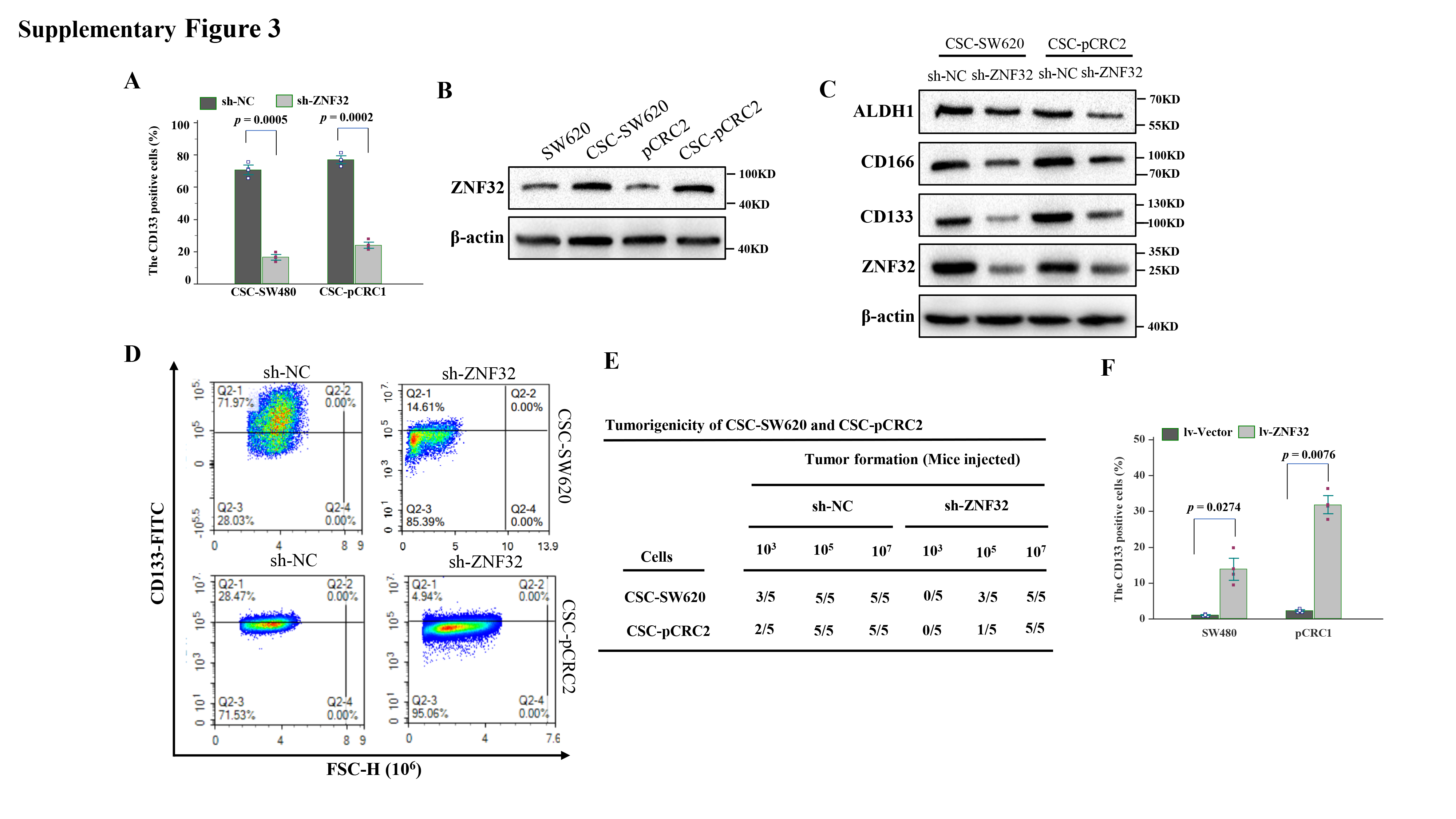

Supplement: Supplementary file 4 — Supplementary figure 3 [file 41419_2022_4530_MOESM4_ESM.tif]

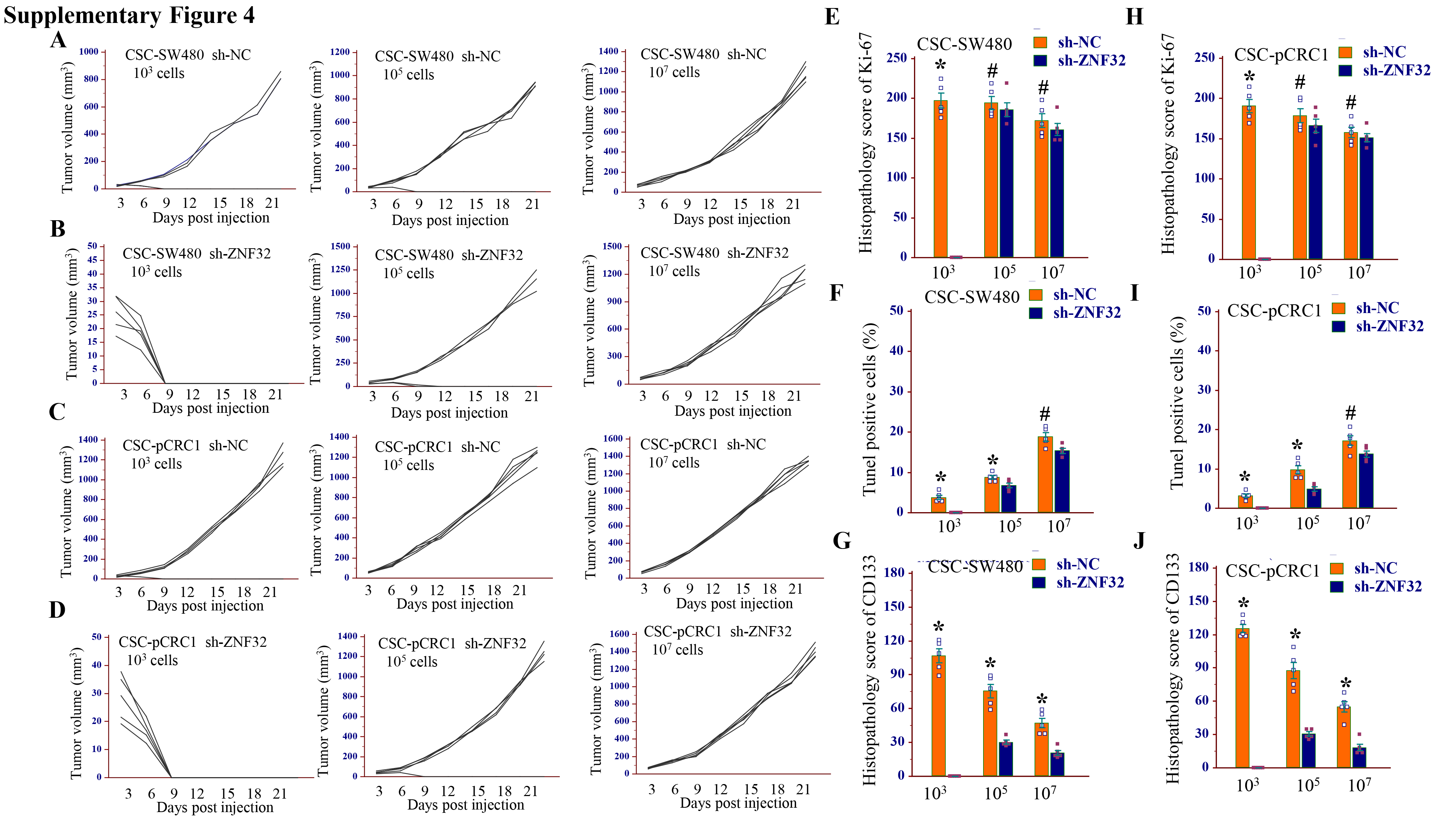

Supplement: Supplementary file 5 — Supplementary figure 4 [file 41419_2022_4530_MOESM5_ESM.tif]

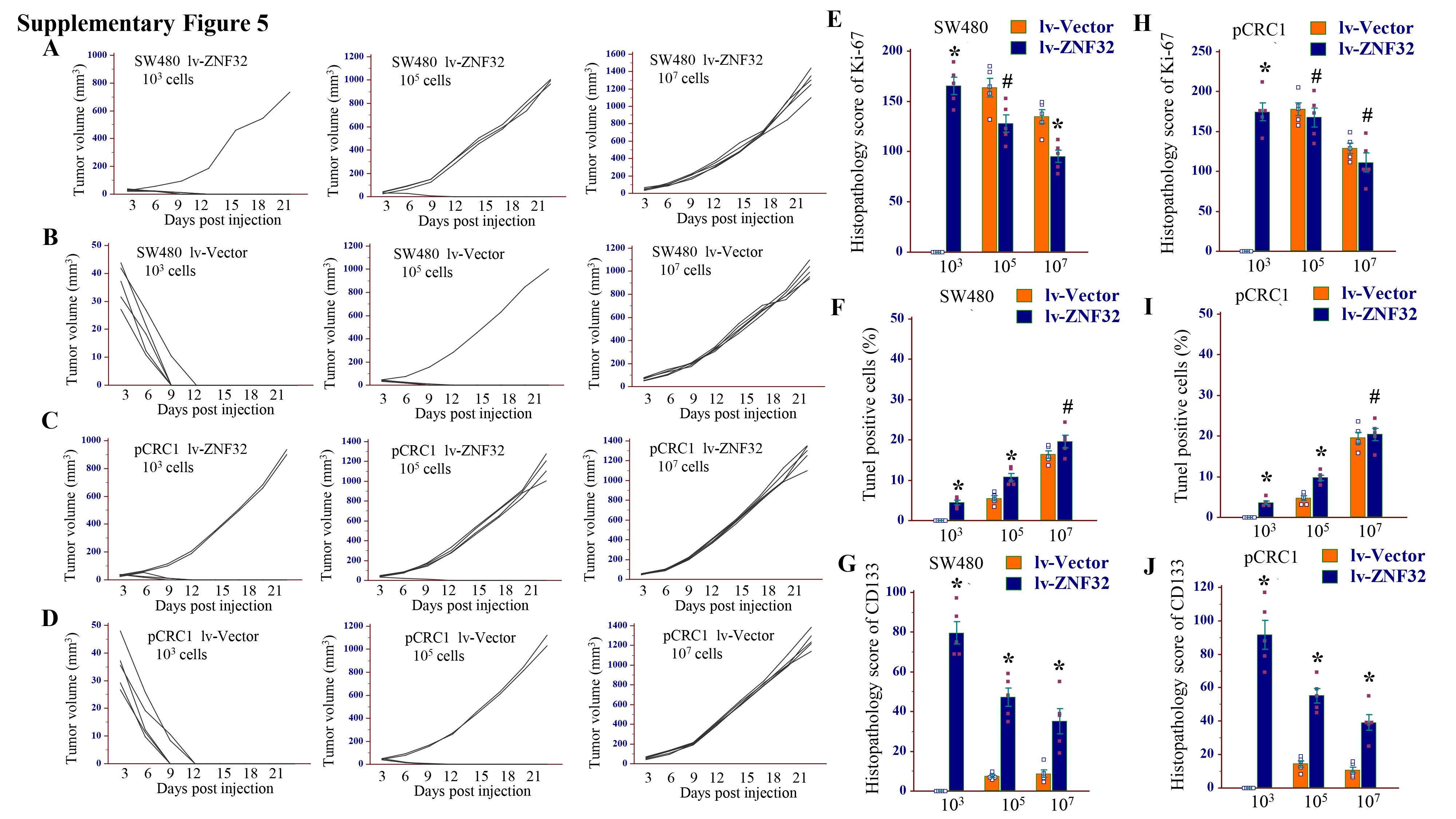

Supplement: Supplementary file 6 — Supplementary figure 5 [file 41419_2022_4530_MOESM6_ESM.tif]

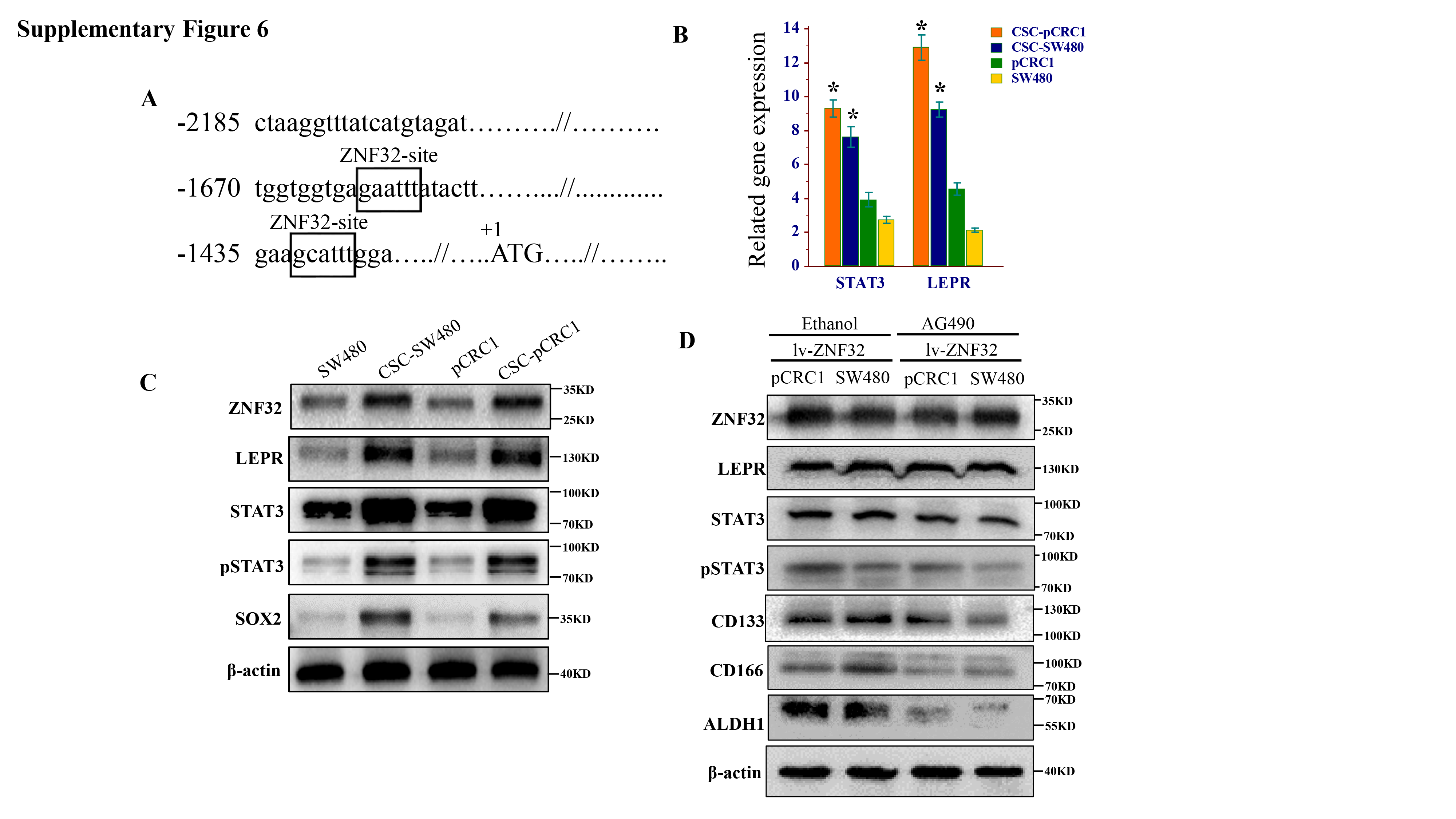

Supplement: Supplementary file 7 — Supplementary figure 6 [file 41419_2022_4530_MOESM7_ESM.tif]

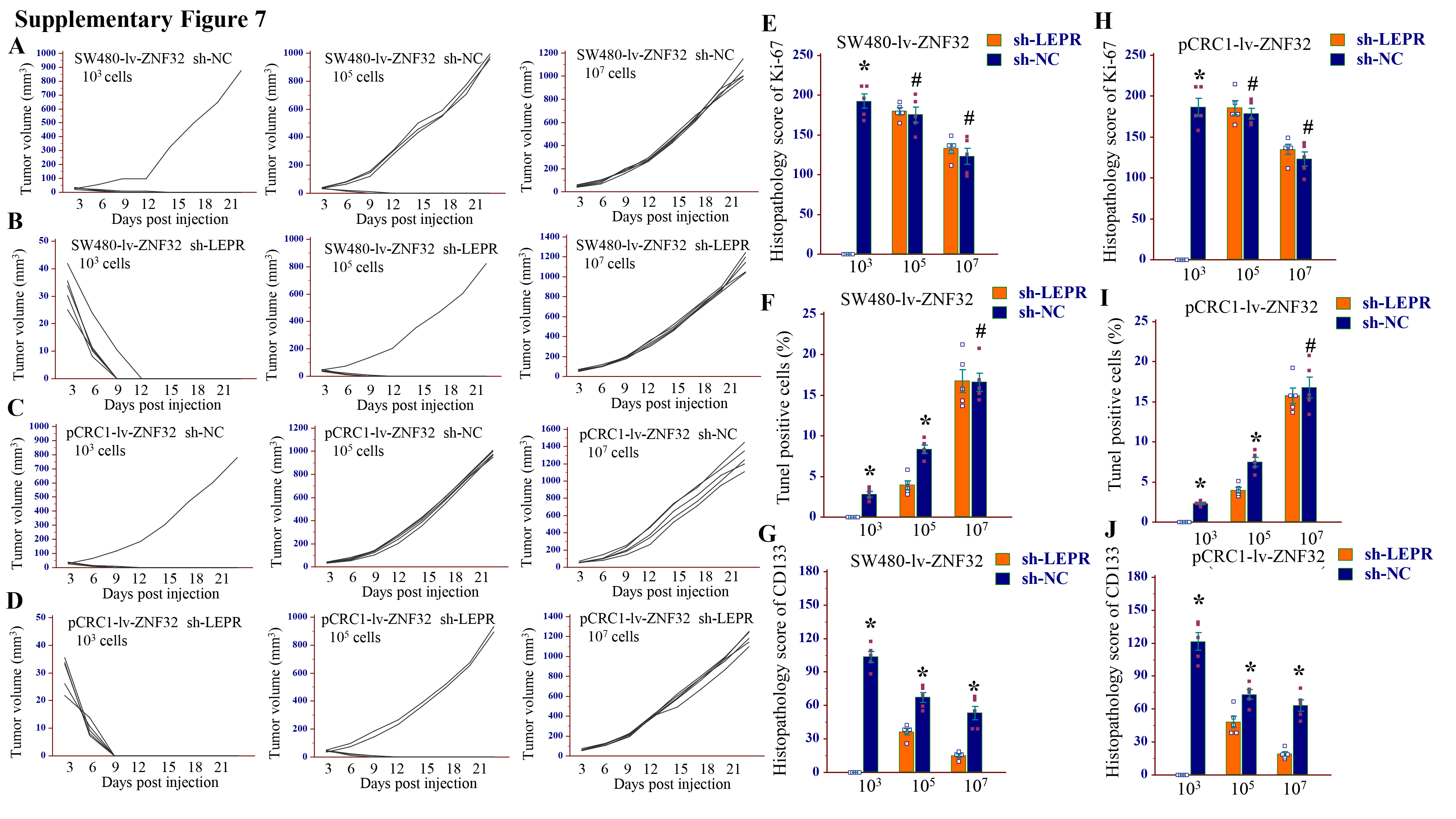

Supplement: Supplementary file 8 — Supplementary figure 7 [file 41419_2022_4530_MOESM8_ESM.tif]

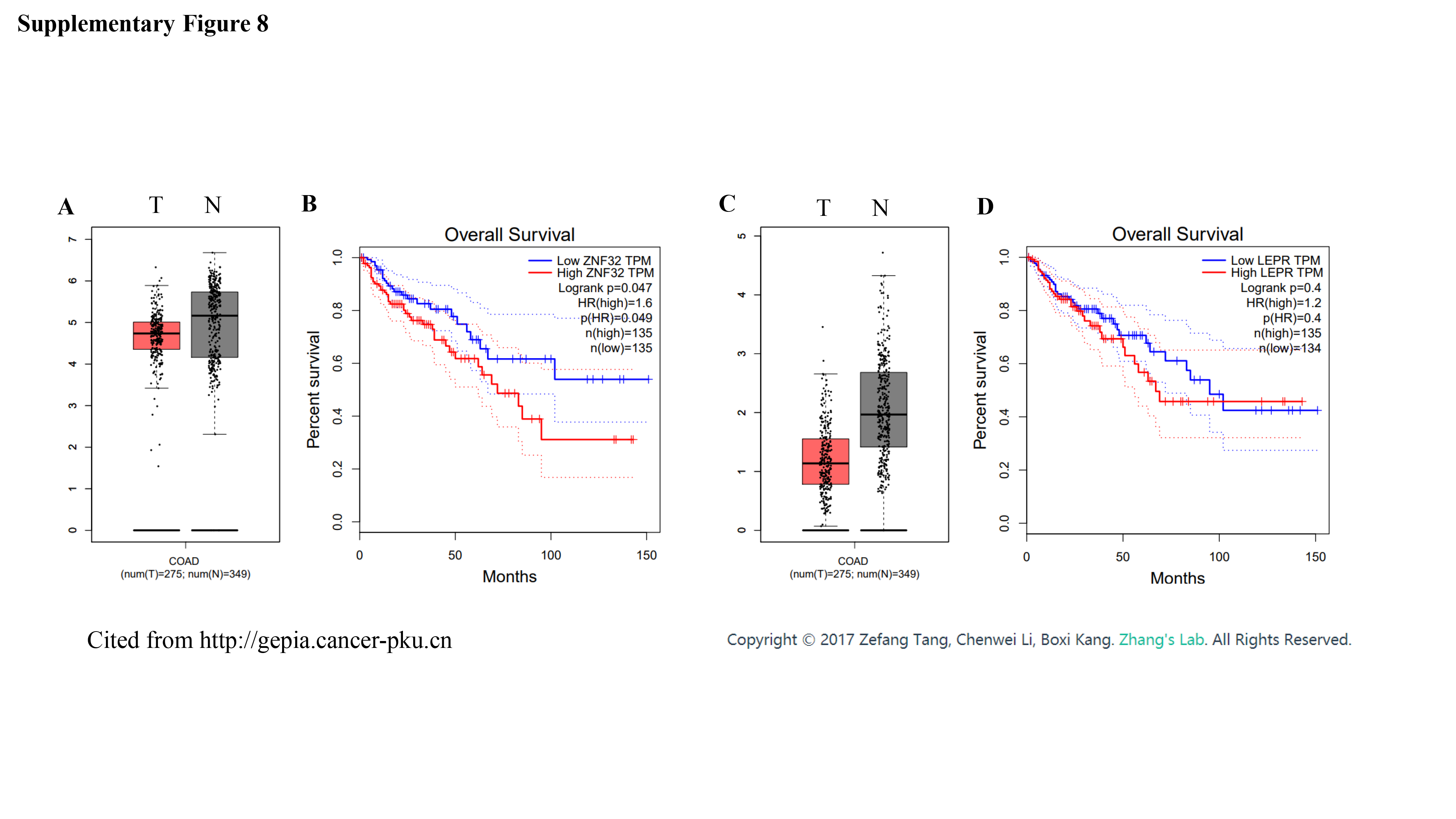

Supplement: Supplementary file 9 — Supplementary figure 8 [file 41419_2022_4530_MOESM9_ESM.tif]
