# Supplementary material for: ZNF32 promotes the self-renewal of colorectal cancer cells by regulating the LEPR-STAT3 signaling pathway
Source: Cell Death Dis. 2022 Feb 3;13(2):108. doi: 10.1038/s41419-022-04530-4 (PMC8814143; doi:10.1038/s41419-022-04530-4)
Supplement: Supplementary file 10 — Supplementary table 1 [file 41419_2022_4530_MOESM10_ESM.docx]

**Supplementary table 1. The genetic status of colon cancer cells.**

**Proficient**

**Positive**

**Mutant**

**Mutant**

**Mutant**

**MMR**

**Mutant**

**Cells**

**BRAF**

**APC**

**TP53**

**KRAS**

**EGFR**

**Proficient**

**Low**

**Mutant**

**Mutant**

**Mutant**

**Mutant**

**SW620**

**SW480**

**HCT116**

**Deficient**

**Positive**

**Mutant**

**Mutant**

**Wild-type**

**Mutant**

**pCRC1**

**Proficient**

**Positive**

**Wild-type**

**Mutant**

**Mutant**

**Wild-type**

**Mutant**

**Wild-type**

**Mutant**

**Mutant**

**Proficient**

**Deficient**

**Low**

**Positive**

**Mutant**

**Mutant**

**Mutant**

**Wild-type**

**pCRC2**

**pCRC3**
