# Supplementary material for: ZNF32 promotes the self-renewal of colorectal cancer cells by regulating the LEPR-STAT3 signaling pathway
Source: Cell Death Dis. 2022 Feb 3;13(2):108. doi: 10.1038/s41419-022-04530-4 (PMC8814143; doi:10.1038/s41419-022-04530-4)
Supplement: Supplementary file 14 — Author changes [file 41419_2022_4530_MOESM14_ESM.docx]

**Description of the author's changes**

Dear editor

I am the corresponding author of the manuscript with the title: “ZNF32 promotes the self-renewal of colorectal cancer cells by regulating the LEPR-STAT3 signaling pathway” (CDDIS-21-0599RRRR). Thank you very much for your work.

In the final manuscript, we added Pro. Xiangsheng Fu and removed Dr. Li Deng. Some adjustments have been made to the order of authors. Because in our study, Dr. Deng Li was originally responsible for RNA-seq and ChIP data analysis, but this work was actually completed by Xiangsheng Fu. At the same time, we changed the strategy of RNA-seq analysis and supplemented a large number of experiments. This work was completed by Pro. Xiangsheng Fu. Therefore, after obtaining the consent of Dr. Deng Li, we agreed that Pro. Xiangsheng Fu was more suitable to enter the author list as the main participant.

In addition, due to the valuable suggestions of the reviewers, we supplemented a large number of experiments to confirm our research, so we also adjusted the author order according to the contributions of different authors.

The above adjustments have been agreed d by all authors. See attachments for related emails.

The author's adjustment is based solely on the author's contribution to this study without any potential conflict of interest.

**Appendix**

| **发件人：LJ <84183967@qq.com>** |  |
| --- | --- |

| **时   间：2022年01月09日（星期日）下午13 : 05** |  |
| --- | --- |
| **收件人：Xiaoan <435445611@qq.com >** |  |
|  |  |

Pro. Xiaoan Li

In our final manuscript of “ZNF32 promotes the self-renewal of colorectal cancer cells by regulating the LEPR-STAT3 signaling pathway”, we changed the order of authors based on their contributions. Since Dr. Li Deng entrusted the RNA-seq and ChIP data analysis to Prof. Xiangsheng Fu. Therefore, we added Prof. Xiangsheng Fu as the main contributing author of the article, and removed Dr. Li Deng. I hope the authors are aware of these changes, and please let me know if you have any suggestions.

Jun Li

| 发件人：Xiaoan  <435445611@qq.com > |  |
| --- | --- |

时   间：2022年01月10日（星期一）上午11 : 05

收件人：JL  <84183967@qq.com >

Dear Jun

I have received your letter. I agree with your changes to the author. I also think it is very important to rank based on actual contributions. I wish you all the best.

Xiaoan

| **发件人：LJ <84183967@qq.com>** |  |
| --- | --- |

| **时   间：2022年01月09日（星期日）下午13 : 05** |  |
| --- | --- |
| **收件人：Lili <1448935641@qq.com >** |  |
|  |  |

Dear Lili

In our final manuscript of “ZNF32 promotes the self-renewal of colorectal cancer cells by regulating the LEPR-STAT3 signaling pathway”, we changed the order of authors based on their contributions. Since Dr. Li Deng entrusted the RNA-seq and ChIP data analysis to Prof. Xiangsheng Fu. Therefore, we added Prof. Xiangsheng Fu as the main contributing author of the article, and removed Dr. Li Deng. I hope the authors are aware of these changes, and please let me know if you have any suggestions.

Jun Li

| 发件人：Lili <1448935641@qq.com > |  |
| --- | --- |

时   间：2022年01月11日（星期二）下午16 : 05

收件人：JL <84183967@qq.com >

Dear Teacher

I agree with you on the change of authors and order. Happy New Year!

Lili

| **发件人：LJ <84183967@qq.com>** |  |
| --- | --- |

| **时   间：2022年01月09日（星期日）下午14 : 00** |  |
| --- | --- |
| **收件人：DL < dengl22@mcmaster.ca >** |  |
|  |  |

Dear Dr.Li

I hope everything goes well with you recently. I need to confirm with you again about the author of the article discussed with you last time. We all agreed that the order of authors based on their contributions.

After discussion with you, the RNA-seq and ChIP data analysis in our study was mainly completed by Professor Fu Xiangsheng. So we added Professor Fu Xiangsheng as the author of the article and deleted your name. Thank you very much for your sincerity and preciseness. I look forward to more cooperation with you. Happy new year to you and your family. Please let us know if you have any questions.

Jun Li

| 发件人：DL < dengl22@mcmaster.ca > |  |
| --- | --- |

时   间：2022年01月12日（星期三）上午03 : 05

收件人：JL <84183967@qq.com >

Dear Dr. Jun

I'm very sorry that COVID-19's epidemic has affected my work in recent two years. At that time, you entrusted to conduct RNA-seq and ChIP data analysis, but due to the work arrangement, it was mainly completed by Professor Fu Xiangsheng. Later, your research needs to change the analysis strategy and supplement a lot of new analysis work. I really don't have more time to complete this part of the work, so ask Professor Fu Xiangsheng continue to complete the follow-up analysis. I think based on his contribution, he should become one of the authors of the article. It's reasonable to delete my name from the author. We look forward to more cooperation in the future.

Li Deng

| **发件人：LJ <84183967@qq.com>** |  |
| --- | --- |

| **时   间：2022年01月09日（星期日）下午14 : 05** |  |
| --- | --- |
| **收件人：SL<1591389941@qq.com >** |  |
|  |  |

Dear Lin

In our final manuscript of “ZNF32 promotes the self-renewal of colorectal cancer cells by regulating the LEPR-STAT3 signaling pathway”, we changed the order of authors based on their contributions. Since Dr. Li Deng entrusted the RNA-seq and ChIP data analysis to Prof. Xiangsheng Fu. Therefore, we added Prof. Xiangsheng Fu as the main contributing author of the article, and removed Dr. Li Deng. I hope the authors are aware of these changes, and please let me know if you have any suggestions.

Jun Li

| 发件人：SL <1591389941@qq.com > |  |
| --- | --- |

时   间：2022年01月10日（星期一）上午9 : 05

收件人：JL <84183967@qq.com >

Dear Teacher

I have no objection to the author change based on his contribution to our research. I wish you all the best in the new year.

Lin Sun

| **发件人：LJ <84183967@qq.com>** |  |
| --- | --- |

| **时   间：2022年01月09日（星期日）下午14 : 10** |  |
| --- | --- |
| **收件人：Xuemei<460782614@qq.com >** |  |
|  |  |

Dear Xuemei

In our final manuscript of “ZNF32 promotes the self-renewal of colorectal cancer cells by regulating the LEPR-STAT3 signaling pathway”, we changed the order of authors based on their contributions. Since Dr. Li Deng entrusted the RNA-seq and ChIP data analysis to Prof. Xiangsheng Fu. Therefore, we added Prof. Xiangsheng Fu as the main contributing author of the article, and removed Dr. Li Deng. I hope the authors are aware of these changes, and please let me know if you have any suggestions.

Jun Li

| 发件人：Xuemei <460782614@qq.com > |  |
| --- | --- |

时   间：2022年01月11日（星期二）上午11 : 00

收件人：JL <84183967@qq.com >

Dear Jun

I agreed that added Prof. Xiangsheng Fu as the main contributing author of the article, and removed Dr. Li Deng.

Xuemei

| **发件人：LJ <84183967@qq.com>** |  |
| --- | --- |

| **时   间：2022年01月09日（星期日）下午14 : 15** |  |
| --- | --- |
| **收件人：Yaqi<2621698550@qq.com >** |  |
|  |  |

Dear Yaqi

In our final manuscript of “ZNF32 promotes the self-renewal of colorectal cancer cells by regulating the LEPR-STAT3 signaling pathway”, we changed the order of authors based on their contributions. Since Dr. Li Deng entrusted the RNA-seq and ChIP data analysis to Prof. Xiangsheng Fu. Therefore, we added Prof. Xiangsheng Fu as the main contributing author of the article, and removed Dr. Li Deng. I hope the authors are aware of these changes, and please let me know if you have any suggestions.

Jun Li

| 发件人：Yaqi< 2621698550@qq.com > |  |
| --- | --- |

时   间：2022年01月10日（星期一）上午10 : 00

收件人：JL <84183967@qq.com >

Dear Teacher

I agree with your amendment to the author. I hope everything goes well .

Yaqi Li

| **发件人：LJ <84183967@qq.com>** |  |
| --- | --- |

| **时   间：2022年01月09日（星期日）下午14 : 15** |  |
| --- | --- |
| **收件人：TY<1530621799@qq.com >** |  |
|  |  |

Dear Yan

In our final manuscript of “ZNF32 promotes the self-renewal of colorectal cancer cells by regulating the LEPR-STAT3 signaling pathway”, we changed the order of authors based on their contributions. Since Dr. Li Deng entrusted the RNA-seq and ChIP data analysis to Prof. Xiangsheng Fu. Therefore, we added Prof. Xiangsheng Fu as the main contributing author of the article, and removed Dr. Li Deng. I hope the authors are aware of these changes, and please let me know if you have any suggestions.

Jun Li

| 发件人：TY< 1530621799@qq.com > |  |
| --- | --- |

时   间：2022年01月10日（星期一）上午9 : 00

收件人：JL <84183967@qq.com >

Dear Jun

I think changed the order of authors based on their contributions. Because Dr. Li Deng entrusted the RNA-seq and ChIP data analysis to Prof. Xiangsheng Fu. I think we should add Prof. Xiangsheng Fu as the main contributing author of the article, and removed Dr. Li Deng.

Tan Yan

| **发件人：LJ <84183967@qq.com>** |  |
| --- | --- |

| **时   间：2022年01月09日（星期日）下午14 : 22** |  |
| --- | --- |
| **收件人：Tongqin<413264001@qq.com >** |  |
|  |  |

Dear Tongqin

In our final manuscript of “ZNF32 promotes the self-renewal of colorectal cancer cells by regulating the LEPR-STAT3 signaling pathway”, we changed the order of authors based on their contributions. Since Dr. Li Deng entrusted the RNA-seq and ChIP data analysis to Prof. Xiangsheng Fu. Therefore, we added Prof. Xiangsheng Fu as the main contributing author of the article, and removed Dr. Li Deng. I hope the authors are aware of these changes, and please let me know if you have any suggestions.

Jun Li

| 发件人：Tongqin< 413264001@qq.com > |  |
| --- | --- |

时   间：2022年01月11日（星期二）上午9 : 30

收件人：JL <84183967@qq.com >

Dear Jun

I agree with your changes to the order of authors and their additions and deletions.

Tongqin Zhang

| **发件人：LJ <84183967@qq.com>** |  |
| --- | --- |

| **时   间：2022年01月09日（星期日）下午14 : 25** |  |
| --- | --- |
| **收件人：ZY<269000947@qq.com >** |  |
|  |  |

Dear Dr.Yan

In our final manuscript of “ZNF32 promotes the self-renewal of colorectal cancer cells by regulating the LEPR-STAT3 signaling pathway”, we changed the order of authors based on their contributions. Since Dr. Li Deng entrusted the RNA-seq and ChIP data analysis to Prof. Xiangsheng Fu. Therefore, we added Prof. Xiangsheng Fu as the main contributing author of the article, and removed Dr. Li Deng. I hope the authors are aware of these changes, and please let me know if you have any suggestions.

Jun Li

| 发件人：ZY< 269000947@qq.com > |  |
| --- | --- |

时   间：2022年01月10日（星期一）上午10: 30

收件人：JL <84183967@qq.com >

Dear Jun

That’s right, I think.

Zhou Yan

| **发件人：LJ <84183967@qq.com>** |  |
| --- | --- |

| **时   间：2022年01月09日（星期日）下午14 : 30** |  |
| --- | --- |
| **收件人：Chunfen<** **mochunfen@cmc.edu.cn >** |  |
|  |  |

Dear Dr.Mo

In our final manuscript of “ZNF32 promotes the self-renewal of colorectal cancer cells by regulating the LEPR-STAT3 signaling pathway”, we changed the order of authors based on their contributions. Since Dr. Li Deng entrusted the RNA-seq and ChIP data analysis to Prof. Xiangsheng Fu. Therefore, we added Prof. Xiangsheng Fu as the main contributing author of the article, and removed Dr. Li Deng. I hope the authors are aware of these changes, and please let me know if you have any suggestions.

Jun Li

| 发件人：Chunfen< mochunfen@cmc.edu.cn > |  |
| --- | --- |

时   间：2022年01月11日（星期一）上午11: 30

收件人：JL <84183967@qq.com >

Dear Jun

It is very correct to rank the authors based on their contributions to the research. At the same time, I admire Dr. Deng Li's honesty. We must thank Professor Fu Xiangsheng for his contribution, so there is no problem with his signature. I wish you all the best!

Mo

| **发件人：LJ <84183967@qq.com>** |  |
| --- | --- |

| **时   间：2022年01月09日（星期日）下午14 :45** |  |
| --- | --- |
| **收件人：Xiangsheng<** **1358088785@qq.com >** |  |
|  |  |

Dear Pro.Fu

Thank you very much for your contribution to this research. Based on the previous communication, we decided to add you as the main contributing author of the article, and removed Dr. Li Deng

I hope the authors are aware of these changes, and please let me know if you have any suggestions.

Jun Li

| 发件人：Xiangsheng< 1358088785@qq.com > |  |
| --- | --- |

时   间：2022年01月12日（星期三）上午8: 30

收件人：JL <84183967@qq.com >

Dear Jun

Thank you very much for your recognition of my work. Because of the influence of COVID-19, Dr Deng Li is hard to find time to complete this part of the analysis. Thank you very much for Dr. Deng Li's recommendation and rigorous research. I have no comment on the author's change.

We look forward to more cooperation in the future

Fu
